# The genomic landscape of breast and non-breast cancers from individuals with germline CHEK2 deficiency

**DOI:** 10.1093/jncics/pkae044

**Published:** 2024-06-07

**Authors:** Snežana Hinić, Rachel S van der Post, Lilian Vreede, Janneke Schuurs-Hoeijmakers, Saskia Koene, Erik A M Jansen, Franziska Bervoets-Metge, Arjen R Mensenkamp, Nicoline Hoogerbrugge, Marjolijn J L Ligtenberg, Richarda M de Voer

**Affiliations:** Department of Human Genetics, Radboud University Medical Center, Research Institute for Medical Innovation, Nijmegen, The Netherlands; Department of Pathology, Radboud University Medical Center, Research Institute for Medical Innovation, Nijmegen, The Netherlands; Department of Human Genetics, Radboud University Medical Center, Research Institute for Medical Innovation, Nijmegen, The Netherlands; Department of Human Genetics, Radboud University Medical Center, Research Institute for Medical Innovation, Nijmegen, The Netherlands; Department of Human Genetics, Radboud University Medical Center, Research Institute for Medical Innovation, Nijmegen, The Netherlands; Department of Human Genetics, Radboud University Medical Center, Research Institute for Medical Innovation, Nijmegen, The Netherlands; Department of Pathology, Radboud University Medical Center, Research Institute for Medical Innovation, Nijmegen, The Netherlands; Department of Human Genetics, Radboud University Medical Center, Research Institute for Medical Innovation, Nijmegen, The Netherlands; Department of Human Genetics, Radboud University Medical Center, Research Institute for Medical Innovation, Nijmegen, The Netherlands; Department of Human Genetics, Radboud University Medical Center, Research Institute for Medical Innovation, Nijmegen, The Netherlands; Department of Pathology, Radboud University Medical Center, Research Institute for Medical Innovation, Nijmegen, The Netherlands; Department of Human Genetics, Radboud University Medical Center, Research Institute for Medical Innovation, Nijmegen, The Netherlands

## Abstract

*CHEK2* is considered to be involved in homologous recombination repair (HRR). Individuals who have germline pathogenic variants (gPVs) in *CHEK2* are at increased risk to develop breast cancer and likely other primary cancers. PARP inhibitors (PARPi) have been shown to be effective in the treatment of cancers that present with HRR deficiency—for example, caused by inactivation of *BRCA1/2*. However, clinical trials have shown little to no efficacy of PARPi in patients with *CHEK2* gPVs. Here, we show that both breast and non-breast cancers from individuals who have biallelic gPVs in *CHEK2* (germline CHEK2 deficiency) do not present with molecular profiles that fit with HRR deficiency. This finding provides a likely explanation why PARPi therapy is not successful in the treatment of *CHEK2*-deficient cancers.

Checkpoint kinase 2 (*CHEK2*) is a tumor suppressor gene and is considered a key component of the DNA damage response and the homologous recombination repair (HRR) of DNA double-strand breaks (1). Individuals heterozygous for germline pathogenic variants (gPVs) in *CHEK2* have a low-to-moderate risk to develop breast cancer (2,3). Individuals with biallelic gPVs in *CHEK2* are, next to breast cancer, likely also at increased risk for other primary cancers (4).

It has been shown that cancers that have a defective HRR (HRD) are sensitive to platinum-based therapies and PARP inhibitors (PARPi) (5). Some PARPi treatment approvals are limited to cancers that have gPVs in *BRCA1/*2 or genomic instability features indicative of HRD (6). It is thought that cancers that are defective for other components of the HRR pathway, such as *ATM, CHEK1, CHEK2, NBN, BRIP1, MRE11, RAD50, RAD51B, RAD51C, RAD51D, RAD54L, PALB2* and *BARD1*, may also benefit from PARPi (7). However, studies have shown that no clinical benefit is seen in individuals who have *ATM* or *CHEK2* gPVs (8).

Recent molecular studies on breast cancers occurring in individuals who are heterozygous for gPVs in *CHEK2* suggest that these cancers do not present with genomic instability features indictive of HRD (9,10). However, these studies focused only on breast cancers from individuals heterozygous for gPVs in *CHEK2.* The aim of this study was to investigate the molecular profiles of cancers from individuals with biallelic gPVs in *CHEK2* (from here on CHEK2-deficient cancers) (11), which may offer explanations why PARPi therapy is ineffective in CHEK2-deficient tumors. We analyzed the genomes, using shallow whole-genome sequencing (sWGS) and whole-exome sequencing (WES), from 16 cancers of 9 individuals homozygous for the most common loss-of-function (LoF) gPV in *CHEK2* (NM_007194.4; c.1100del) (12). In total, we analyzed 8 breast cancers and 8 other CHEK2-deficient cancers [colorectal cancer (CRC) (n =* *4), thyroid (n = 2), endometrial (n = 1), and urothelial (n = 1) cancer].

We compared our findings to breast (n =* *3) and non-breast cancers (n =* *25) from individuals heterozygous for *CHEK2* gPVs and to breast (n =* *7) and non-breast cancers (n =* *21) from individuals heterozygous for *BRCA1/2* gPVs (13). All, except 2 cancers with a heterozygous *CHEK2* gPV, did not harbor a somatic second hit. The cancers from individuals heterozygous for gPVs in *BRCA1/2* were selected for having a second somatic hit rendering them BRCA1/2-deficient (Supplementary Table 1, available online). To investigate tumor mutational profiles, we analyzed genomic instability features including large-scale state transitions (LST) and telomeric allelic imbalances (tAI). Furthermore, we analyzed tumor mutational burden (TMB), mutational signatures via SigProfiler (14,15) and putative cancer driver gene mutations (Supplementary Figure 1, available online). To determine the differences in the presence of somatic pathogenic variants (PVs) in driver genes and the presence of mutational signatures between groups, a Fisher exact test was used. For comparisons of more than 2 groups, one-way analysis of variance or Kruskal-Wallis test were applied. All statistical tests are two-sided, and statistical significance level is a *P* value less than .05. Further details are provided in the Supplementary Methods (available online).

The presence of LSTs and tAIs are genomic instability features indicative of HRD (16). The median number of LSTs and tAIs in CHEK2-deficient cancers was 3 (range = 1-12) and 7 (range = 0-22), respectively (Figure 1, A and B; Supplementary Figure 2, available online). None of the analyzed cancers presented with an LST count of 15 or more, which is considered the cutoff value for HRD. An additional in-house reference set of BRCA1/2-deficient ovarian cancers (n = 5) presented with a median number of LSTs and tAIs of 23 (range = 16-31) and 16 (range = 12-18), respectively (Figure 1, A and B; Supplementary Figure 2, available online). From the Cancer Genome Atlas (TCGA), we also retrieved the LST and tAI counts of cancers from individuals who are heterozygous for *CHEK2* gPVs or *BRCA1/*2 gPVs. The median LST and tAI counts in cancers from individuals heterozygous for *CHEK2* gPVs were 4 (range = 0-16) and 8.5 (range = 0-26), respectively, which is comparable to the counts observed in our CHEK2-deficient cancers (Figure 1, A and B; Supplementary Figure 2, available online). BRCA1/2-deficient cancers, as expected, presented with higher LST and tAI counts (median LST count of 26 [range = 12-39], with a median tAI count of 24 [range = 17-32]), (Figure 1, A and B; Supplementary Figure 2, available online). Our findings are in line with literature on breast cancers with heterozygous *CHEK2* (g)PVs (10) and show no statistically significant differences in genomic instability features between breast and non-breast cancers of CHEK2-deficient individuals (Supplementary Figure 3, A and B, available online).

**Figure 1.**
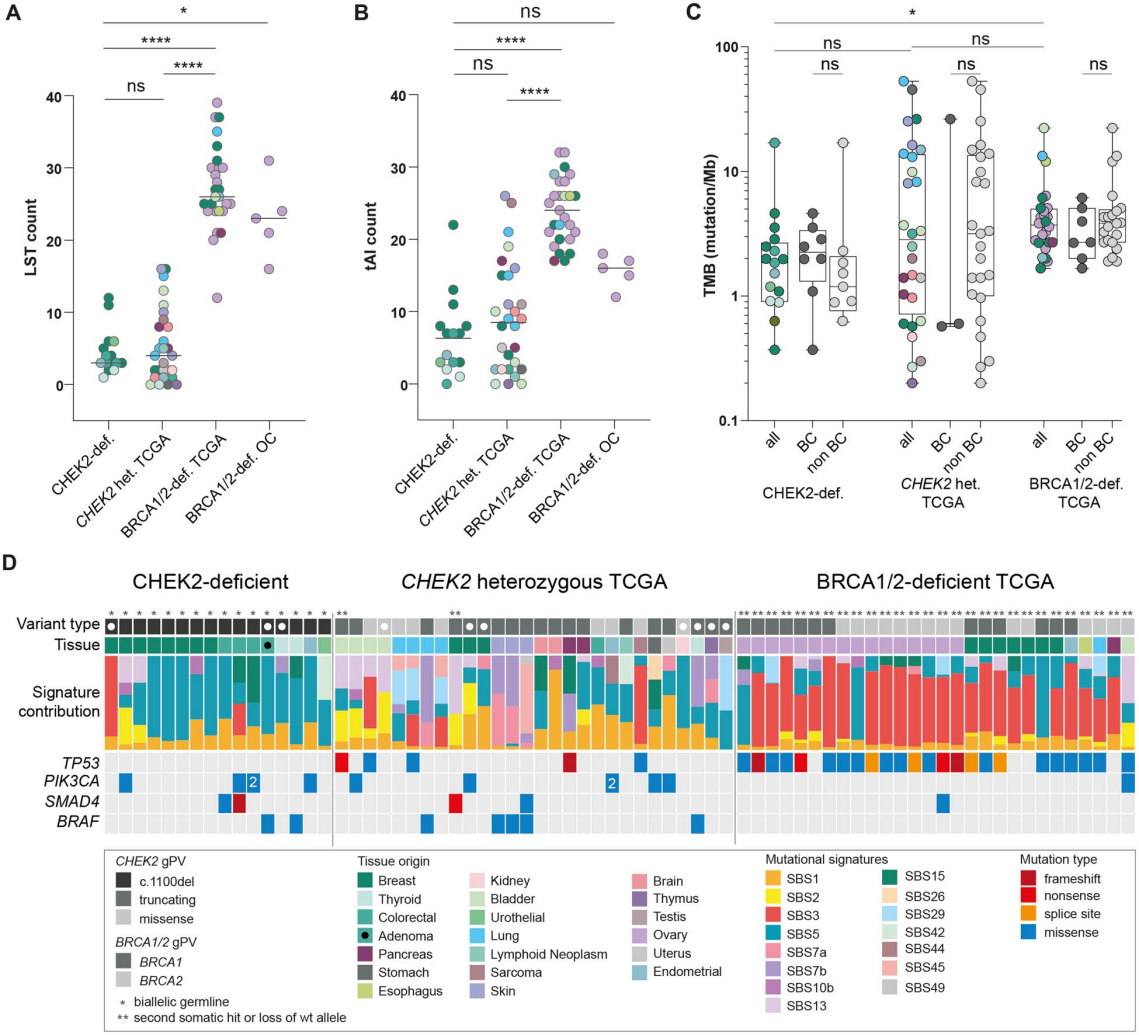
The mutational landscape of CHEK2-deficient cancers. Genomic instability features including **A**) large-scale state transitions (LST) counts and **B**) telomeric allelic imbalances (tAI) counts are depicted. Groups (left to right) included in-house generated data for CHEK2-deficient cancers; in-house generated data for BRCA1/2-deficient ovarian cancers; the Cancer Genome Atlas (TCGA) data of cancers from individuals heterozygous for *CHEK2* germline pathogenic variants (gPVs); and TCGA data of BRCA1/2-deficient cancers. Cancer origins are represented in different colors. Lines represent median values per group. Groups were compared via nonparametric Kruskal-Wallis test, and Dunn’s multiple comparison correction was applied. *P < .05; ****P < .0001. **C**) Tumor mutational burden (TMB) of nonsynonymous variants in all cancers and between breast and non-breast cancers are depicted. CHEK2-deficient cancers are compared to the cancers from individuals heterozygous for *CHEK2* gPVs from TCGA and BRCA1/2-deficient cancers from TCGA. Lines represent the median value per group. Groups were compared via nonparametric Kruskal-Wallis test, and Dunn’s multiple comparison correction was applied. *P < .05. **D**) CHEK2-deficient cancers, cancers from individuals heterozygous for *CHEK2* gPVs from TCGA, and BRCA1/2-deficient cancers from TCGA were analyzed for single base substitution mutational signature contributions and mutated driver genes. The black dot labels one premalignant adenomatous colonic polyp and white dots indicate cancer samples with <30 single base substitutions, which indicates that mutational signatures from these samples are not completely reliable. gPV = germline pathogenic variant; TCGA = The Cancer Genome Atlas; TMB = tumor mutational burden; LST = large-scale state transitions; tAI = telomeric allelic imbalances; ns = nonsignificant; BC = breast cancer; non-BC = non-breast cancer; OC = ovarian cancer; wt = wild-type; het = heterozygous; def = deficient.

Next, we investigated the nonsynonymous TMB and microsatellite instability (MSI). The median TMB was 1.92 (range = 0.37-16.99 variants/Megabase) in CHEK2-deficient cancers, which is comparable to cancers that developed in individuals heterozygous for *CHEK2* gPVs (median TMB of 2.83 [range = 0.2-53 variants/Megabase]; Figure 1, C) and in line with the literature (9,10). BRCA1/2-deficient cancers presented with a significantly higher TMB than CHEK2-deficient cancers (median TMB of 3.72 [range = 1.67-22.27 variants/Megabase]; *P *< .02; Figure 1, C). Furthermore, the TMB was not significantly different between breast and non-breast CHEK2-deficient cancers (*P* = .2786; Figure 1, C). One CHEK2-deficient cancer, a colorectal cancer (CRC), showed MSI.

Subsequently, we analyzed mutational signatures. Most CHEK2-deficient cancers presented with the clock-like mutational signatures SBS1 (5/16 cancers; 31%) and/or SBS5 (14/16 cancers; 88%; Figure 1, D, Supplementary Table 1, available online). Cancers from individuals heterozygous for *CHEK2* gPVs presented with mutational signatures SBS1 and/or SBS5 in 12/28 (43%; *P *=* *1) and 12/28 (43%; *P* < .05) cancers, respectively (Figure 1, D). In contrast to the CHEK2-deficient cancers, BRCA1/2-deficient cancers did not present with mutational signature SBS1 (0/28 cancers; 0%; *P* < .05), and a minority presented with mutational signature SBS5 (11/28 cancers; 39%; *P* < .05; Figure 1, D). SBS3, the HRD-related mutational signature (17), was observed to some level in 13% (2/16) of CHEK2-deficient cancers, and it was the predominant mutational signature (86%, 24/28) in the BRCA1/2-deficient group (*P* < .001; Figure 1, D). This finding is in concordance with current literature on breast cancers from individuals heterozygous for *CHEK2* PVs (9,10) and indicates that HRD does not seem to be driving the process of tumorigenesis in CHEK2-deficient cancers. In 25% (4/16) of CHEK2-deficient cancers, tissue-specific signatures, including mutational signature SBS15 (defective DNA-mismatch repair) in two CRCs (1 with somatic *MSH2* inactivation) and mutational signatures SBS2 and SBS13 (APOBEC-related signatures) in 2 breast cancers, were identified (Figure 1, D). Cancers from individuals heterozygous for *CHEK2* gPVs also presented with tissue-specific mutational signatures in 10/28 (36%) cancers, including mutational signatures SBS2 and SBS13 in bladder and breast cancers, and mutational signature SBS29 (tobacco chewing) in lung cancers (Figure 1, D).

We further compared mutational signatures occurring in the CHEK2-deficient cancers to those in a cohort of (mostly) sporadic cancers (n = 7515) from TCGA of various tissue origins (Supplementary Figure 4, available online). The spectrum of mutational signatures in CHEK2-deficient cancers resembles sporadic cancers more than what is observed in BRCA1/2-deficient cancers (Supplementary Figure 4, available online), which is also observed when we compare CHEK2-deficient cancers with cancers with heterozygous gPV in *BRCA1/2*, for which it was not clear if a somatic second hit was present (n =* *134 cancers). The latter group has a prominent contribution of mutational signature SBS3, similar to BRCA1/2-deficient cancers, whereas this is absent in CHEK2-deficient cancers (Supplementary Figure 4, available online). Next to the observed tissue-specific mutational signatures, breast and non-breast CHEK2-deficient cancers present with comparable mutational signature profiles (Supplementary Figure 3, C, available online). Our data are not in support of a single base mutational signature that can be linked to CHEK2 deficiency, as has been shown for *BRCA1/2* and other genes involved in DNA repair, such as the mismatch repair genes or genes involved in base excision repair (17).

Last, we investigated the mutated cancer driver genes in cancers from CHEK2-deficient individuals. The most frequently mutated gene in CHEK2-deficient cancers was *PIK3CA* (n = 4 cancers, three cancer types; Figure 1, D). No somatic PVs were observed in *TP53* in CHEK2-deficient cancers (Figure 1, D; Supplementary Figure 3, D, available online), in line with Smid et al. (9), whereas this was the most frequently mutated gene in BRCA1/2-deficient cancers (0/16 vs 24/28; *P* < .001; Figure 1, D). Furthermore, although a complete absence of somatic *TP53* PVs was observed in breast cancers with heterozygous *CHEK2* gPVs (n =* *3), non-breast cancers with heterozygous *CHEK2* gPVs did present with somatic *TP53* PVs (Figure 1, D; Supplementary Figure 3, D, available online). This finding suggests that either *TP53* somatic PVs are not necessary for cancer development in the context of *CHEK2*-deficiency or that concurrent inactivation of both *CHEK2* and *TP53* does not confer a growth benefit for cells. In line with this, *CHEK2* was identified to cause synthetic lethality in an in silico study on *TP53* druggable target partners (18). Although *TP53* is one of the most important tumor suppressor genes that also transmits DNA-damage-induced signals received from both *ATM* and *CHEK2*, *CHEK2* also operates in an independent manner from the HRR pathway in cell-cycle arrest, apoptosis, and senescence (1).

In summary, our data show that CHEK2-deficient cancers are not driven by disruption of HRR as genomic instability features and a mutational signature indicative of HRD are lacking. The absence of a CHEK2-associated mutational signature suggests that *CHEK2* does not play a prominent role in DNA repair mechanisms. Its role in regulation of cell division may be more important, which is in line with the apparent mutual exclusivity of CHEK2 deficiency and somatic *TP53* mutations.

Our study has a few limitations. We were limited in structural variant size analysis, as the resolution of our sWGS is lower than the WGS approach that was used by Smid et al. and, therefore, does not permit us to investigate structural variants smaller than 1 Mb (9). Furthermore, we were only able to investigate the molecular profile for CHEK2-deficient cancers of 4 origins beyond breast cancer. Ideally, this should be expanded to more cancer types occurring in individuals who have biallelic *CHEK2* gPVs and to more cancers per type.

Taken together, our study provides additional evidence that impairment of *CHEK2* does not result in HRD, which likely explains the inefficiency of PARPi treatment of CHEK2-deficient cancers.

## Supplementary Material

pkae044_Supplementary_Data

## Data Availability

All WES and sWGS data generated in this study are available from European Genome-Phenome Archive (EGA) numbers EGAS50000000080 and EGAS00001007258 via https://ega-archive.org/. Public data are available through https://www.cbioportal.org/.
