# Supplementary material for: The genomic landscape of breast and non-breast cancers from individuals with germline CHEK2 deficiency
Source: JNCI Cancer Spectr. 2024 Jun 7;8(4):pkae044. doi: 10.1093/jncics/pkae044 (PMC11216722; doi:10.1093/jncics/pkae044)
Supplement: pkae044_Supplementary_Data [file pkae044_supplementary_data.zip › JNCICS-24-0030R1_Supplementary_materials_20.3.24.pdf]

Supplementary Material:

The genomic landscape of breast- and non-breast cancers from individuals with germline CHEK2-deficiency

Snežana Hinić, MSc[1], Rachel S. van der Post, PhD[2], Lilian Vreede, BSc[1], Janneke Schuurs-Hoeijmakers, PhD[1], Saskia Koene, PhD[1], Erik A.M. Jansen, BSc[1], Franziska Bervoets-Metge, PhD[2], Arjen R. Mensenkamp, PhD[1], Nicoline Hoogerbrugge, PhD[1], Marjolijn J.L. Ligtenberg, PhD[1,2], Richarda M. de Voer, PhD[1]

Affiliations:

[1]Radboud university medical center, Research Institute for Medical Innovation, Department of Human Genetics, Nijmegen, the Netherlands

[2]Radboud university medical center, Research Institute for Medical Innovation, Department of Pathology, Nijmegen, the Netherlands

Supplementary Methods

Study cohort

This study included cancers from individuals identified to be homozygous for the *CHEK2* c.1100del germline pathogenic variant (gPV) at the Radboudumc, Nijmegen, the Netherlands. All individuals either had an early-onset breast cancer (before the age of 50 years, *n* = 3) or developed multiple primary malignancies (*n* = 6). Information on cancers was requested through the Dutch Nationwide Pathology Databank (PALGA)[1] and available cancers were obtained for sequencing. Written informed consent was obtained and this study was approved by the local medical ethics committee of the Radboud university medical center (CMO; study numbers 2019-5082, 2019-5738 and 2020-7036).

Control cohorts

i) The Cancer Genome Atlas (TCGA)[2] germline datasets that consisted of cancer samples with gPVs in either *BRCA1*, *BRCA2* or *CHEK2* (*n* = 190) were collected[3]. From this dataset, cancer samples from individuals with a gPV in *BRCA1* or *BRCA2*, and somatic loss of the wild-type allele or second somatic hit in the cancer (BRCA1/2-deficient; *n* = 28) were used as positive controls for defective homologous recombination repair (HRD; **Supplementary Table 1**). Cancer samples from individuals heterozygous for *CHEK2* gPVs were used as heterozygous *CHEK2* controls (*n* = 28; **Supplementary Table 1**). Two samples had a second somatic hit (a bladder cancer with a somatic pathogenic variant (PV) and a breast cancer with a loss of the wild-type allele), while 26 samples only had a gPV in *CHEK2* and no somatic inactivation (**Supplementary Table 1**). The remaining samples were from individuals that had a gPV in either *BRCA1* or *BRCA2*, but not a

somatic second hit and it was unclear if there was a loss of the wild-type allele in the cancer (*n* = 134). The data was obtained under the TCGA data request number #106777-1.

ii) TCGA PanCancer Atlas Studies somatic data (*n* = 7,515) consisted of cancer samples that matched tissue origins observed in cancers from individuals with gPVs in either *CHEK2*, *BRCA1* or *BRCA2* and included the following cancer origins (from 21 PanCancer studies): breast, ovary, bladder, head and neck, thyroid, esophagus, colorectum, stomach, endometrium, uterus, brain (low grade glioma and glioblastoma), lung (adenocarcinoma and squamous cell carcinoma), pancreas, sarcoma, skin, B cell lymphoma, kidney (chromophobe), testicular and thymus. All publicly available somatic variants were downloaded from cBioPortal and were part of the PanCancer TCGA project originally deposited on the NCI Genomic Data Commons[2,4-6].

iii) In-house ovarian cancer samples with gPVs in *BRCA1* or *BRCA2* were used as a positive control when comparing copy number alteration (CNA) profiles (*n* = 5; **Supplementary Table 1**).

More extensive information including histological (sub)type and cancer stage, where available, can be found in the **Supplementary Table 1**. Additional information on the data types used from each cohort can be found in **Supplementary Figure 1**.

Pathology review of CHEK2-deficient cancers

All available formalin-fixed paraffin-embedded (FFPE) tumor blocks (*n* = 16 cancers from 9 individuals) were reviewed by an expert pathologist. We included 15 cancers and one pre-malignant adenomatous polyp. H&E stainings were reviewed by a pathologist to identify neoplastic cells. Cancer cell percentage and histological (sub)type(s) were collected (**Supplementary Table 1**).

Whole exome sequencing and bioinformatic processing

Genomic DNA (gDNA) was extracted from FFPE cancer slides (*n* = 16; **Supplementary Table 1**) and used to generate whole-exome libraries using the Agilent SureSelectXT Human All Exon v7 enrichment (Agilent Technologies, Santa Clara, CA) (*n* = 15) and Twist Bioscience enrichment (*n* = 1) kits according to the manufacturer's instructions. Subsequently, exome libraries where sequenced on a NovaSeq 6000 (Illumina) with 2x150bp paired-end reads. After demultiplexing, sequence reads where aligned to the reference genome (version GRCh37) using BWA version 0.7.13[7]. Somatic variants were called using MuTect2 V4.0.6.0[8] and annotated using an in-house developed annotation pipeline[9]. High-confident somatic variants with >10% variant allele frequency and at least 15-read coverage and ≥ 4 reads supporting the variant allele were selected for further analysis. Furthermore, any variants present in our in-house database of germline variation, the Genome Aggregation Database (gnomAD) or present >2 times in the Exome Aggregation Consortium database (ExAC) were excluded[10]. To ensure FFPE induced artefacts were removed, variants with <4 reads supporting the variant allele were excluded and variants with <2 reads supporting the variant allele per read pair were also removed. Furthermore, when >1 cancer sample from an individual was subjected to whole exome sequencing, all prioritized variants were compared between cancer samples from the same individual and potential germline variants were excluded. To ensure variant validity, randomly chosen variants were manually checked using the Integrative Genome Viewer[11].

Shallow whole genome sequencing and copy number alteration analysis

To investigate the genome-wide CNA, gDNA extracted from cancer tissues (*n* = 15) was subjected to shallow whole-genome sequencing (sWGS) on the NovaSeq 6000 (Illumina) with 2x150 bp paired-end reads. The median coverage was 1.5x [range: 0.9-2.1x] per genome. Ovarian cancer samples with a heterozygous gPV in either *BRCA1* or *BRCA2* (*n* = 5) were used as positive controls

for HRD hallmark CNA profiles and sequenced as aforementioned. Cancer sWGS data were assessed for quality and demultiplexed via an in-house pipeline and alignment was performed to the reference genome GRCh38 using BWA (version 2.2.1). To call and investigate the CNA, ichorCNA[12] with a 1 Megabase bin size and ploidies of 2, 3 and 4 was used, after which the best ploidy fit was determined and used for further analyses in each sample. Indicator measurements for genomic instability were investigated including: i) Large-scale state transitions (LST) that include genomic imbalances per chromosome arm of ≥10 Megabases in size, and ii) telomeric allelic imbalances (tAI) that include genomic imbalances at the telomeric ends of ≥2 Megabases in size, but do not span the centromere. Counting, comparisons and visualizations were performed in R v3.6 using RStudio. Data obtained from the TCGA was compared to our dataset (**Supplementary Figure 1**).

Tumor mutational burden and microsatellite instability analysis

To analyze molecular cancer features, non-synonymous tumor mutational burden was calculated taking into account all regions covered ≥15x (total variant count/Megabase). Furthermore, to investigate microsatellite instability (MSI) status, 152 MSI loci were analyzed the CHEK2-deficient cancers. For samples from the TCGA, MSI Mantis score was obtained with a cutoff of 0.6.

Mutational signature analysis

SigProfiler MatrixGenerator was used to create mutational matrices based on single base substitution (SBS) 96-trinucleotide mutational profiles[13]. To create *de novo* mutational signatures and decompose them into known mutational signatures available at the Catalogue of Somatic Mutations in Cancer (COSMIC) (v3.2, 2021)[14], SigProfiler Extractor was used[15]. Mutational signatures were extracted under the default parameters set by SigProfiler Extractor. The following groups were used as controls for the mutational signature analysis: i) a mixed dataset of somatic variants from samples

that carried a gPV in either *BRCA1*, *BRCA2* or *CHEK2* (n = 190)[3] and ii) a mixed dataset of somatic variants that matched cancer types that we sequenced and cancer types that matched samples carrying gPVs in the following genes: *CHEK2*, *BRCA1* and *BRCA2* (n = 7,515; Supplementary Figure 1). Mutational signature contributions of ≥20% were considered relevant and were investigated in further detail. In cancer samples with less than 30 SBS, mutational signatures extracted were considered not to be reliable, due to the low number of somatic variants. To visualize different mutational signature profiles, SigProfiler Plotting and R package ggplot2 were used[13,16]. All analyses were done in R v3.6 using RStudio[17,18].

Analysis of somatic variants in cancer driver genes

To assess the occurrence of somatic PVs in putative cancer driver genes, somatic PVs were prioritized based on two lists of cancer driver genes: the OncoKB database[19] and a manually curated list of driver genes (n = 299 genes) identified via a PanCancer TCGA analyses[20]. The overlapping mutated genes that were identified in our dataset were filtered in a following manner: i) variants absent from our in-house germline database were prioritized, ii) variants that were present in EXAC database[21] ≤2 times were prioritized, iii) variants absent from gnomAD-genomes were prioritized[10]. This approach ensured that only rare somatic variants were included in the following analysis. Additionally, we prioritized only non-synonymous coding variants and genes that were mutated in ≥2 cancers in at least two of the investigated groups. For comparison, we performed the same analysis on the control datasets from the TCGA (Supplementary Figure 1).

Statistical analysis

P values, odds ratio (OR) and 95% confidence intervals (CI) were calculated using R software v4.1. Bonferroni correction for multiple testing was applied and results were labeled as statistically

significant when $P < .05$. For comparisons of more than two groups, one-way analysis of variance followed by Tuckey's post-hoc test or Kruskal-Wallis test followed by Dunn's multiple comparison test (for non-normally distributed data) were applied in GraphPad Prism version 9.3.1.

References

1. Casparie M, Tiebosch AT, Burger G, et al. Pathology databanking and biobanking in The Netherlands, a central role for PALGA, the nationwide histopathology and cytopathology data network and archive. *Cell Oncol*. 2007;29(1):19-24. doi:10.1155/2007/971816

2. The Cancer Genome Atlas (TCGA). https://www.cancer.gov/about-nci/organization/ccg/research/structural-genomics/tcga

3. Huang KL, Mashl RJ, Wu Y, et al. Pathogenic Germline Variants in 10,389 Adult Cancers. *Cell*. Apr 5 2018;173(2):355-370.e14. doi:10.1016/j.cell.2018.03.039

4. Cerami E, Gao J, Dogrusoz U, et al. The cBio cancer genomics portal: an open platform for exploring multidimensional cancer genomics data. *Cancer Discov*. May 2012;2(5):401-4. doi:10.1158/2159-8290.Cd-12-0095

5. Grossman RL, Heath AP, Ferretti V, et al. Toward a Shared Vision for Cancer Genomic Data. *N Engl J Med*. Sep 22 2016;375(12):1109-12. doi:10.1056/NEJMp1607591

6. Gao J, Aksoy BA, Dogrusoz U, et al. Integrative analysis of complex cancer genomics and clinical profiles using the cBioPortal. *Sci Signal*. Apr 2 2013;6(269):pl1. doi:10.1126/scisignal.2004088

7. Li H, Durbin R. Fast and accurate short read alignment with Burrows-Wheeler transform. *Bioinformatics*. Jul 15 2009;25(14):1754-60. doi:10.1093/bioinformatics/btp324

8. McKenna A, Hanna M, Banks E, et al. The Genome Analysis Toolkit: a MapReduce framework for analyzing next-generation DNA sequencing data. *Genome Res*. Sep 2010;20(9):1297-303. doi:10.1101/gr.107524.110

9. portal HGss. Web Annotation for HCDiffs and VCF. 2023. http://turbo-l4:8444/WebAnnotation/

10. Karczewski KJ, Francioli LC, Tiao G, et al. The mutational constraint spectrum quantified from variation in 141,456 humans. *Nature*. 2020/05/01 2020;581(7809):434-443. doi:10.1038/s41586-020-2308-7

11. Robinson JT, Thorvaldsdóttir H, Winckler W, et al. Integrative genomics viewer. *Nat Biotechnol*. Jan 2011;29(1):24-6. doi:10.1038/nbt.1754

12. Adalsteinsson VA, Ha G, Freeman SS, et al. Scalable whole-exome sequencing of cell-free DNA reveals high concordance with metastatic tumors. *Nature Communications*. 2017/11/06 2017;8(1):1324. doi:10.1038/s41467-017-00965-y

13. Bergstrom EN, Huang MN, Mahto U, et al. SigProfilerMatrixGenerator: a tool for visualizing and exploring patterns of small mutational events. *BMC Genomics*. Aug 30 2019;20(1):685. doi:10.1186/s12864-019-6041-2

14. Tate JG, Bamford S, Jubb HC, et al. COSMIC: the Catalogue Of Somatic Mutations In Cancer. *Nucleic Acids Res*. Jan 8 2019;47(D1):D941-d947. doi:10.1093/nar/gky1015

15. Islam SMA, Díaz-Gay M, Wu Y, et al. Uncovering novel mutational signatures by de novo extraction with SigProfilerExtractor. *Cell Genomics*. 2022/11/09/ 2022;2(11):100179. doi:https://doi.org/10.1016/j.xgen.2022.100179

16. Wickham H. *ggplot2: Elegant Graphics for Data Analysis*. Springer-Veerlag; 2016.

17. *RStudio: Integrated Development for R: RStudio, PBC*. 2020. http://www.rstudio.com

18. *R: A language and environment for statistical computing. R Foundation for Statistical Computing: R Foundation for Statistical Computing*. 2017. https://www.R-project.org

19. Chakravarty D, Gao J, Phillips SM, et al. OncoKB: A Precision Oncology Knowledge Base. *JCO Precis Oncol*. Jul 2017;2017doi:10.1200/po.17.00011

20. Bailey MH, Tokheim C, Porta-Pardo E, et al. Comprehensive Characterization of Cancer Driver Genes and Mutations. *Cell*. Apr 5 2018;173(2):371-385.e18. doi:10.1016/j.cell.2018.02.060

21. Lek M, Karczewski KJ, Minikel EV, et al. Analysis of protein-coding genetic variation in 60,706 humans. *Nature*. 2016/08/01 2016;536(7616):285-291. doi:10.1038/nature19057

Supplementary Tables

Supplementary Table 1 – large excel file (attached separately)

Supplementary Figures

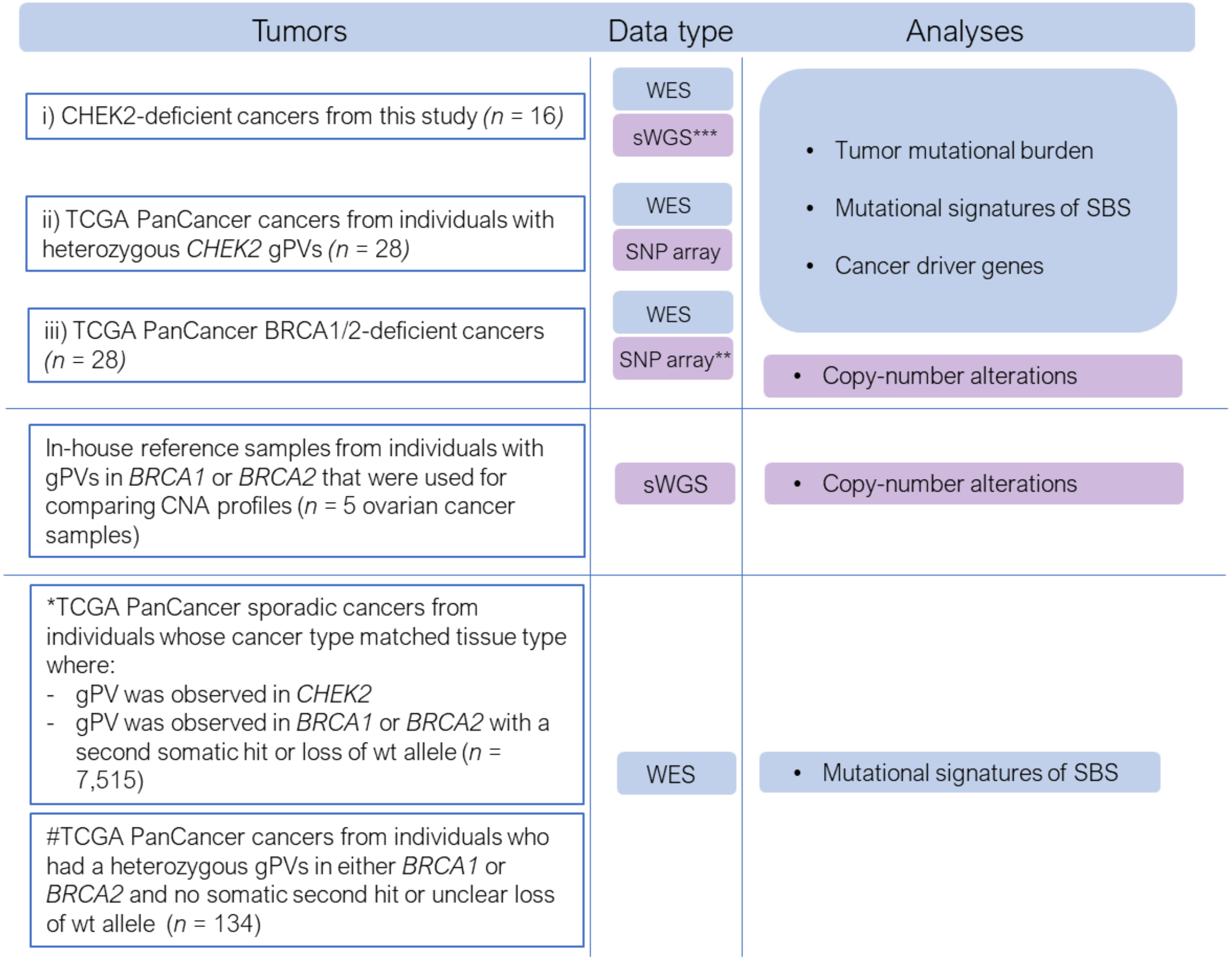

Supplementary Figure 1: Scheme of all data resources and data types included in this study. On the left, data sources are listed including the in-house generated sequencing data and data obtained from The Cancer Genome Atlas (TCGA). Data type denotes which data were used from each data resource. Analyses types are color coded according to the data type used. Asterisk denotes cancer origins from 21 PanCancer studies: breast, ovary, bladder, head and neck, thyroid, esophagus, colorectum, stomach, endometrium, uterus, brain

(low grade glioma and glioblastoma), lung (adenocarcinoma and squamous cell carcinoma), pancreas, sarcoma, skin, B cell lymphoma, kidney (chromophobe), testis and thymus. Two asterisks denote that SNP array data were available for 27/28 BRCA1/2-deficient cancers. Three asterisks denote that sWGS data were available for 15/15 CHEK2-deficient cancers. # denotes cancer origins from 23 PanCancer studies: breast, cervix, ovary, bladder, head and neck, thyroid, esophagus, colorectum, stomach, endometrium, brain (glioblastoma), lung (adenocarcinoma and squamous cell carcinoma), liver, pancreas, prostate, sarcoma, skin, kidney (clear cell and papillary cell carcinoma), testis, pheochromocytoma and paraganglioma, thymus. Abbreviations: SBS, single base substitutions; sWGS; shallow whole genome sequencing; wt, wild-type; gPV, germline pathogenic variant; CNA, copy number alteration; WES, whole exome sequencing; SNP, single nucleotide polymorphism; TCGA, The Cancer Genome Atlas.

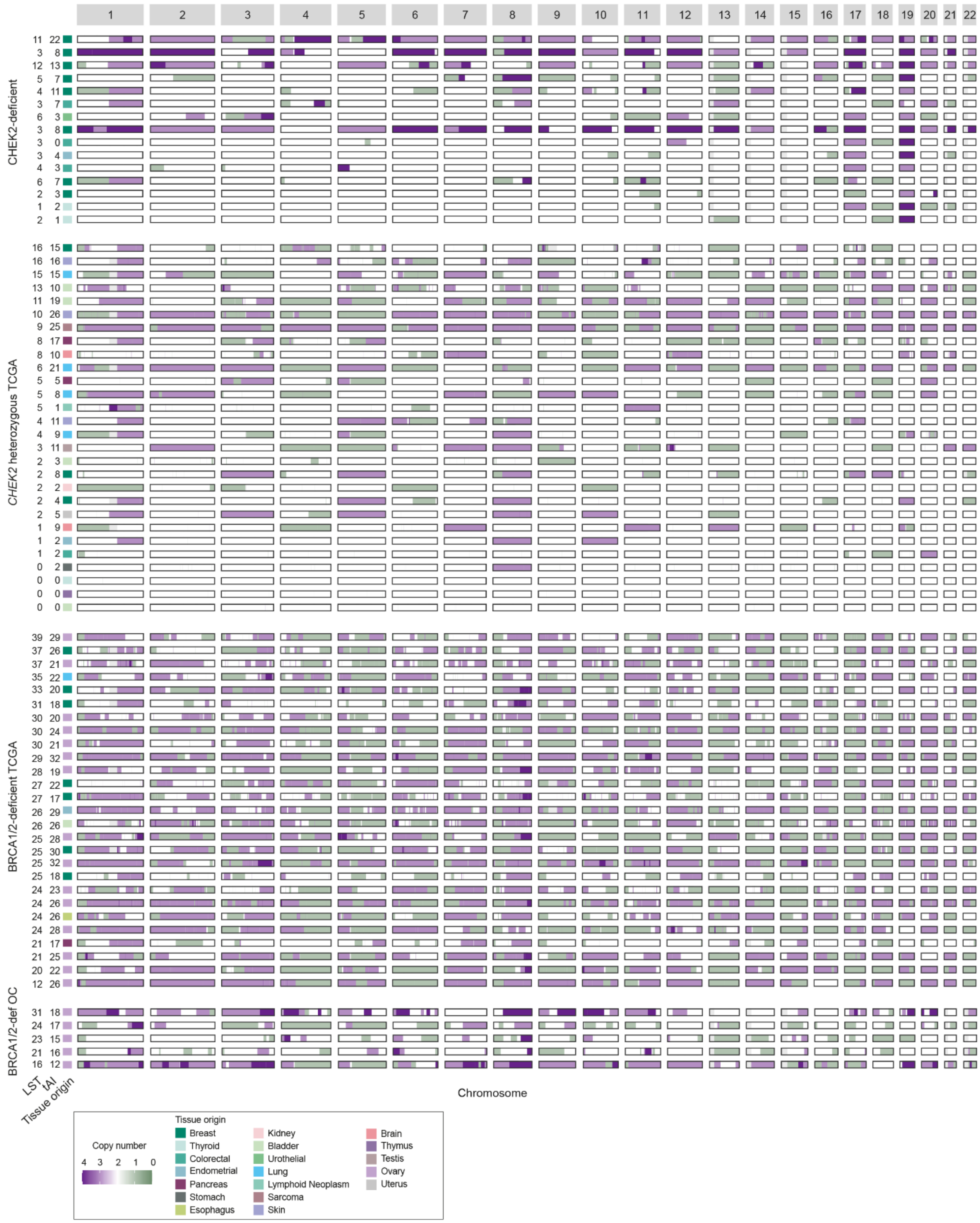

Supplementary Figure 2: Copy number alteration profiles of cancers with gPVs in *CHEK2*, *BRCA1* or *BRCA2*.

Shallow whole genome sequencing was performed on CHEK2-deficient cancers and compared to cancer samples from individuals heterozygous for *CHEK2* gPVs and BRCA1/2-deficient cancers from The Cancer

Genome Atlas (TCGA). Additionally, sWGS was performed on BRCA1/2-deficient ovarian cancers (BRCA1/2-def OC). Genome-wide copy number alterations are depicted in different colors representing deletion (green), gain (purple) and amplification (dark purple). Large-scale state transitions and telomeric allelic imbalances counts are shown on the left. Abbreviations: gPV, germline pathogenic variant; TCGA, The Cancer Genome Atlas; LST, large-scale state transition; tAI, telomeric allelic imbalance; OC, ovarian cancer; def, deficient.

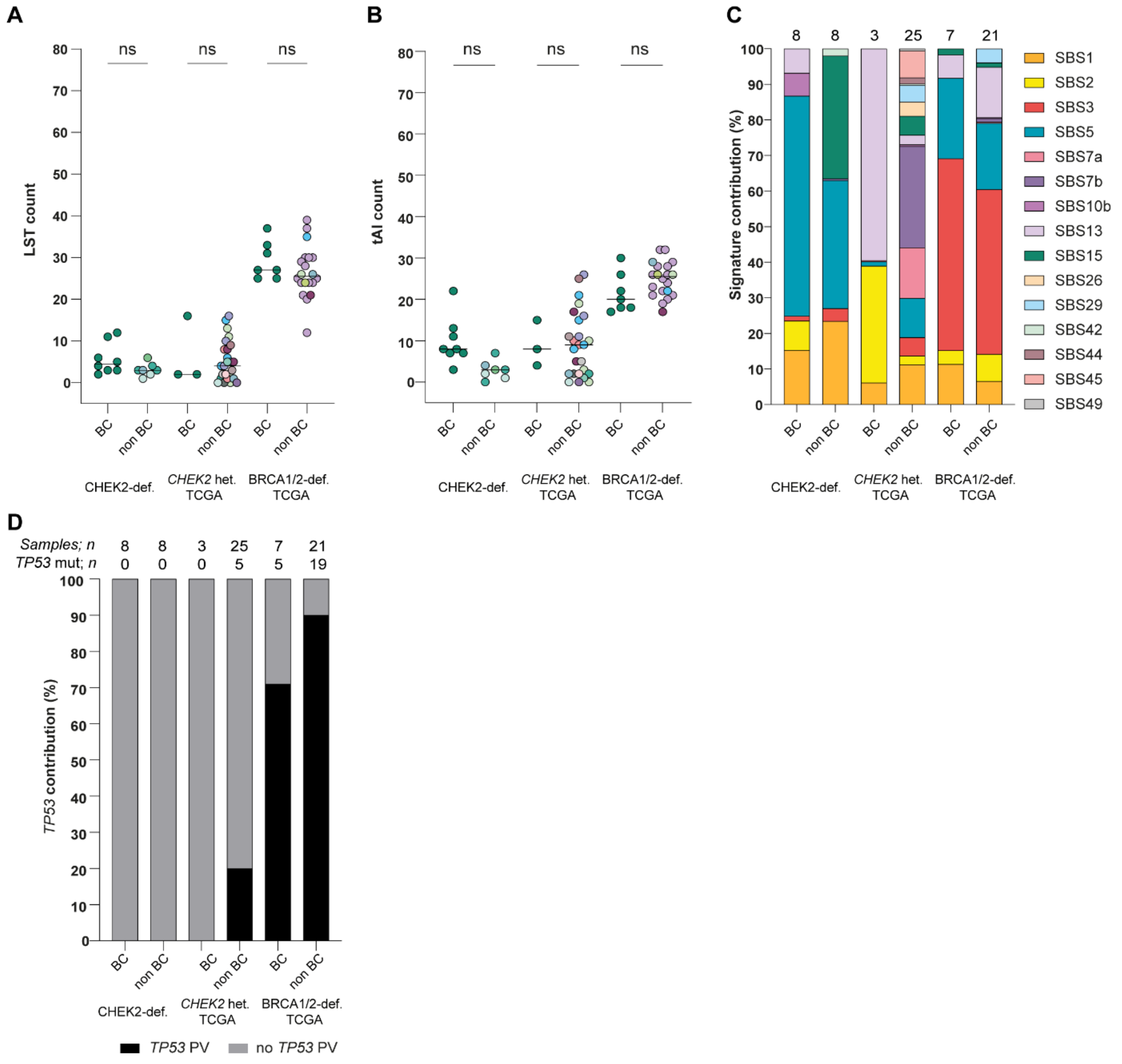

Supplementary Figure 3: Breast- and non-breast cancer molecular landscape comparisons. Breast- and non-breast cancers were compared in groups (left to right) of CHEK2-deficient cancers, cancers from individuals heterozygous for *CHEK2* gPVs and BRCA1/2-deficient cancers. **A)** Large-scale state transitions counts and **B)**

Telomeric allelic imbalance counts in breast and non-breast cancers in the different groups are compared. Comparisons were made via non-parametric Kruskal-Wallis test and Dunn's multiple comparison correction was applied. ns, non-significant. C) Single base substitution mutational signature contributions in breast and non-breast cancers in the different groups are shown. Every single base substitution mutational signature is labeled in a different color. Numbers above the bars represent the number of cancers per group. D) Contribution of somatic pathogenic variants in *TP53* in breast and non-breast cancers in different cancer groups. Numbers of samples represent the number of cancers per group. Number of somatic pathogenic variants (PV) in *TP53* are the number of cancers with a somatic PV in *TP53*. Abbreviations: gPV, germline pathogenic variant; TMB, tumor mutational burden; SBS, single base substitution; LSTs, large-scale state transitions; tAIs, telomeric allelic imbalances; BC, breast cancer; non-BC, non-breast cancer; TCGA, The Cancer Genome Atlas; mut., mutations.

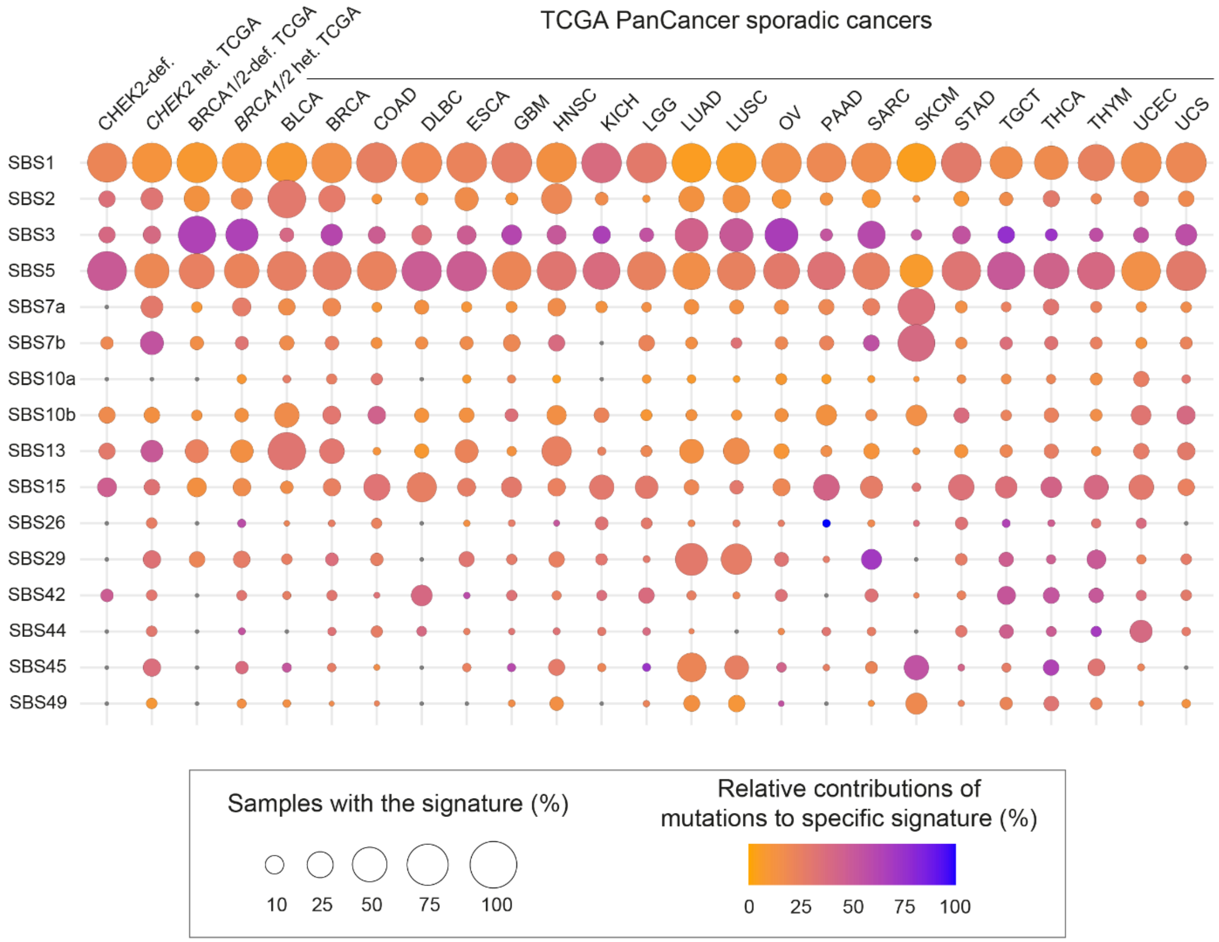

Supplementary Figure 4: Mutational signature contributions in cancers with gPV in *CHEK2*, *BRCA1* or *BRCA2* and sporadic cancers. Relative contribution and the abundance of the single base substitution mutational signatures from the Catalogue of Somatic Mutations in Cancer (COSMIC) are depicted in the bubble plot. CHEK2-deficient cancers, cancers with heterozygous gPVs in *CHEK2,* BRCA1/2-deficient cancers, cancers with heterozygous germline pathogenic variants (gPV) in *BRCA1* or *BRCA2* with unknown deficiency status and sporadic cancers from The Cancer Genome Atlas (TCGA) of different origins are depicted. The *BRCA1/2* heterozygous TCGA group represents cancers from TCGA dataset with heterozygous gPVs and no somatic second hit or when it is unclear if the wild-type allele was lost in the cancer. Abbreviations: SBS, single base substitution; COSMIC, Catalogue Of Somatic Mutations In Cancer; gPV, germline pathogenic variant; TCGA, The Cancer Genome Atlas; Cancer origins: BLCA, bladder; BRCA, breast; COAD, colorectum; DLBC, diffuse large B-cell lymphoma; ESCA, esophagus; GBM, glioblastoma; HNSC, head and neck squamous cell; KICH, kidney (chromophobe); LGG, low-grade glioma; LUAD, LUSC, lung (adenocarcinoma and squamous cell carcinoma, respectively); OV, ovary; PAAD, pancreas; SARC, sarcoma; SKCM, skin; STAD, stomach; TGCT, testis; THCA, thyroid; THYM, thymus; UCEC, endometrium; UCS, uterus.
